# Future Screening Prospects for Ovarian Cancer

**DOI:** 10.3390/cancers13153840

**Published:** 2021-07-30

**Authors:** Diana Žilovič, Rūta Čiurlienė, Rasa Sabaliauskaitė, Sonata Jarmalaitė

**Affiliations:** 1 Life Sciences Center, Institute of Biosciences, Vilnius University, Saulėtekio Avenue 7, LT-10222 Vilnius, Lithuania; 2 Laboratory of Clinical Oncology, National Cancer Institute, Santariškių 1, LT-08406 Vilnius, Lithuania; sonata.jarmalaite@nvi.lt; 3 Oncogynecology Department, National Cancer Institute, Santariškių 1, LT-08406 Vilnius, Lithuania; ruta.ciurliene@nvi.lt; 4 Laboratory of Genetic Diagnostic, National Cancer Institute, Santariškių 1, LT-08406 Vilnius, Lithuania; rasa.sabaliauskaite@nvi.lt

**Keywords:** ovarian cancer, liquid biopsies, uterine lavage, high-throughput methods, NGS-based multigene panels

## Abstract

**Simple Summary:**

Ovarian cancer (OC) has the highest mortality rate of all gynecological cancers. It is usually diagnosed in late stages (FIGO III-IV), and therefore, overall survival is very poor. If diagnosed at the early stages, ovarian cancer has a 90% five-year survival rate. Liquid biopsy has a good potential to improve early ovarian cancer detection and is discussed in this review.

**Abstract:**

Current diagnostic tools used in clinical practice such as transvaginal ultrasound, CA 125, and HE4 are not sensitive and specific enough to diagnose OC in the early stages. A lack of early symptoms and an effective asymptomatic population screening strategy leads to a poor prognosis in OC. New diagnostic and screening methods are urgently needed for early OC diagnosis. Liquid biopsies have been considered as a new noninvasive and promising method, using plasma/serum, uterine lavage, and urine samples for early cancer detection. We analyzed recent studies on molecular biomarkers with specific emphasis on liquid biopsy methods and diagnostic efficacy for OC through the detection of circulating tumor cells, circulating cell-free DNA, small noncoding RNAs, and tumor-educated platelets.

## 1. Introduction

Ovarian cancer (OC) has the highest mortality rate of all gynecologic malignancies [1]. The overall five-year survival is 46% and varies depending on the stage and histological type of the tumor [2]. High-grade serous carcinoma (HGSOC) accounts for 75% of all epithelial ovarian malignancies and is diagnosed mainly at FIGO stage III (51%) or IV (29%), reflecting the aggressive nature [3]. In contrast, nonepithelial and more rare epithelial tumors such as endometrioid, mucinous. and clear-cell carcinomas are more frequently diagnosed at FIGO stages I–II [3]. Consequently, the five-year survival for HGSOC is 43%, compared with 82%, 71%, and 66% for endometrioid, mucinous, and clear-cell carcinoma, respectively. The five-year OS rate is only 9% for FIGO stage IV HGSOC patients [1].

Risk factors for OC can be categorized into genetic and nongenetic risk factors associated with reproductive history, exogenous hormone use, medical history, lifestyle, and environmental influence [4]. It is well established that a family history of OC, especially if a relative was diagnosed under the age of 50, germline *BRCA1/BRCA2* mutations, are the strongest risk factor for this pathology. Lynch syndrome-associated mutations in *MSH2, MSH6, MLH1, PMS2,* and *EPCAM*, as well as mutations in *BRIP1, PALB2, RAD51C,* and *RAD51D* increase the risk for OC. It is considered that approximately 18% of epithelial cancers, in particular high-grade serous carcinomas, are caused by inherited mutations [5,6].

Currently, conventional tools for diagnosing OC are serum cancer antigen 125 (CA 125), Human Epididymis Protein 4 (HE4), and transvaginal ultrasonography (TU). According to the majority of studies, the routine use of CA 125 alone is not adequate for differential diagnosis, as it might increase in other conditions [7,8]. A number of large prospective studies reported that CA 125 ± TU is not sensitive and specific enough for early OC diagnostics, detecting only 30 to 45% of OC in the early stages [7,9,10,11,12]. The use of this approach for OC screening did not demonstrate a survival benefit, and the high rate of false-positive results leads to unnecessary surgery in cancer-free women and is not recommended in the general population due to the potential harms outweighing the potential benefits. An exception could be made for patients with a heredity predisposition to ovarian cancer [7,13,14]. The recent UKCTOS study suggested that in order to improve OC survival rates, we need to reduce the incidence of stage III by more than 10% [15]. Therefore, the development of more sensitive and specific methods for diagnosing OC at the earliest possible stage of the disease would be likely to impact the outcome.

Until recently, OC classification was based on morphology and immunohistochemistry (IHC), but more modern diagnostic approaches take into account molecular genetics, protein post-translational transformations, and immune cell infiltrates [16,17]. Over the last few decades, two distinct pathogenesis models were defined dividing ovarian malignancies into ovarian-origin OC and extra ovarian-origin OC. Ovarian-origin malignancies are very rare, mostly occurring at a young age or in childhood, and are presented by two main groups: (1) sex-cord stromal tumors tend to manifest as low-grade disease with a nonaggressive clinical course and are usually diagnosed at the early stages; (2) predominantly malignant germ cell tumors stand out due to their very fast tumor growth and the progression of clinical symptoms. Therefore, detailed screening tests do not seem mandatory for this category of tumors. The majority of epithelial ovarian cancers (EOCs) and epithelial–stromal ovarian tumors are suspected to be of extra ovarian origin, as the derivative cell is not ovarian (serous, mucinous, endometrioid, clear cell, and others). For clinical decision making, surface epithelial malignancies were further divided into two categories as a function of their pathogenetic pathways: type I and type II [2,18,19,20].

Most malignant tumors of the ovary are surface epithelial (90%). In 2014, the World Health Organization (WHO) recognized five principal epithelial OC histotypes: high-grade serous, low-grade serous, endometrioid, clear cell, and mucinous carcinoma. Other malignancies such as carcinosarcoma, adenosarcoma, and endometrioid stromal sarcoma are very rare; therefore, there is very little data concerning their pathogenesis and molecular features. Moreover, not otherwise specified ovarian tumors such as neuroendocrine, rete ovarii adenocarcinoma, Wilm‘s tumor, and others are exceptionally rare with an incidence of less than 0.1%. The most frequent mutation characteristics according to tumor morphology are presented in Table 1 [18,19,20,21,22,23,24,25,26,27,28,29,30,31].

**Figure 1:**
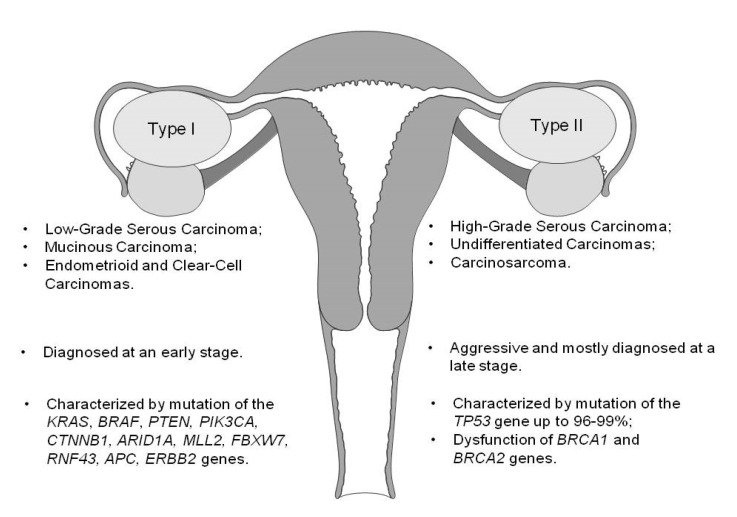
A schematic representation of type I and type II EOC.

Predominantly type I tumors clinically present as large cystic masses, without ascites, tend to be less aggressive, grow more slowly, and usually are diagnosed at an early stage. They are relatively genetically stable, as well as characterized by mutations in different genes (Figure 1) and rarely harbor *TP53* mutations [18,19,20].

Type II tumors are characterized as highly aggressive neoplasms accounting for 75% of all EOCs, which are usually diagnosed at a late stage. They include high-grade serous carcinoma (HGSOC)—the most common type—and rare types such as high-grade endometrioid, undifferentiated carcinomas, and malignant epithelial mesenchymal tumors (carcinosarcomas). Type II ovarian tumors have a high level of genetic instability; the majority harbors *TP53* mutations [18,19,20]. Recent data suggest that HGSOC tumors originate from the epithelium of the fallopian tube. Mutation of *TP53* is the first known molecular event in the transformation of fallopian tube secretory cells to serous tubal intraepithelial carcinomas (STICs), which leads to HGSOC initiation. Mutated *TP53* can be identified as an early tumor precursor of HGSOC. It has been estimated that it takes approximately seven years from STIC to clinically evolve into HGSOC [18,29,32]. Almost 80% of women present with advanced (stages III-IV) disease and poor prognosis (the five-year survival rate is around 25%). Since up to 98% of all HGSOC cases are characterized by *TP53* somatic mutations, this biomarker is widely investigated as a potential diagnostic tool for OC diagnostics [18,20,29,31].

## 2. Materials and Methods

We performed a literature search in NCBI PubMed from January 2014 to September 2020 with a specific emphasis on liquid biopsy biomarkers for early OC detection. We used the keywords “ovarian cancer” together with “circulating free DNA”, ”circulating tumor DNA”, ”circulating tumor cells”, “small non coding RNA”, “microRNA”, “PIWI- interactingRNA”, “Transfer-RNA-derivated small RNA”, “liquid biopsy”, “TEPS”, and “uterine lavage”. We identified 2193 abstracts in NCBI PubMed and selected 30 reports considered inclusion criteria—evaluating the efficacy of liquid biopsies as a diagnostic tool for OC detection. We summarize the results of these studies in Table 2. This work provides deeper understanding of the aspects of OC pathogenesis and existing challenges for liquid biopsy applications in clinical practice.

## 3. Modern Means for Early Detection of OC 

The essential aspect of any screening test is that it should be cost-effective and easily incorporated into standard medical practice. Furthermore, an ideal test should be reproducible, no-invasive, and able to distinguish between a healthy woman and a patient at an early stage of disease. High specificity to avoid false positives and a sensitivity of at least 75% are needed [61]. Due to the prevalence of <1% of OC in the population, a positive predictive value (PPV) of 10% should be obtained for a cost-effective screening tool. This explains the need for a 75% sensitivity and a 99.6% specificity for early-stage disease. New, potentially promising early diagnostic tools can be based on the fact that the ovarian surface, the fallopian tube, and the uterine cavity form a communicating space. The peristaltic waves of the fallopian tube allow the transport of exfoliated cells from HGSCs or STICs into the uterine cavity and peritoneum. Several different ways can be applied for collecting cancer cells from the Müllerian duct: cytological specimens or uterine lavage samples. Sequencing of *TP53* exons is widely performed due to the fact that the majority of HGSOCs are characterized by TP53 mutations [44,62].

### 3.1. Uterine Cavity Lavage Biomarkers

An approach for the lavage of the uterine cavity to detect cancer cells that have been shed was developed by Paul Speiser, Professor at Medical College of Vienna, and colleagues [63].

A study published by Kinde et al. in 2013 analyzed the liquid Pap test from the uterine cervix for detecting ovarian and uterine cancers. Massively parallel sequencing for tumor-specific mutations using a 12-gene panel was performed on DNA extracted from liquid Pap smear tests. This technique was successfully applied to 100% of patients. Detectible DNA mutations were found in 24 (100%) for endometrial cancer patients and in 9 of 22 (41%) OC, mainly in late stages [45]. A pilot study showed that tumor cells and fragments containing tumor DNA can be found and collected in the vagina using a vaginal tampon and studied by using genetic analysis. They succeeded in revealing *TP53* mutations in 60% of advanced HGSOCs [44]. Y. Wang et al. 2018 published data of DNA analysis in Pap brush samples from 245 OC patients, and the detection sensitivity was 33%, including 34% for patients with stage I–II disease [34].

In 2015, E. Maritschnegg et al. collected samples closer to the ovaries and fallopian tubes; uterine cavity lavage was used from 65 patients: 30 with OC, 27 with benign gynecological disease, and 8 with other malignancies. The lavage technique was applied successfully, and sufficient amounts of DNA were obtained. Lavage and tumor specimens were analyzed by massive parallel sequencing. Amplicons comprised gene regions of: *AKT1, APC, BRAF, CDKN2A, CTNNB1, EGFR, FBXW7, FGFR2, KRAS, NRAS, PIK3CA, PIK3R1, POLE, PPP2R1A, PTEN,* and *TP53.* Mutations, mainly in *TP53*, were detected in 60% of OC lavage samples using next-generation sequencing (NGS) approaches. Using additional methods with higher sensitivity, such as digital droplet polymerase chain reaction (ddPCR) and the Safe-sequencing system (SafeSeqS), the mutation detection rate was increased up to 80%. Moreover, *TP53* mutation in lavage samples was identified in all patients (*N* = 5) with FIGO stage IA and one patient with occult OC. Mutations in *KRAS* were identified (eight of twenty-seven cases) in a benign tumor group mainly, and none of them had the *TP53* mutation [43].

Later, in 2018, E. Maritschnegg published data on the uterine lavage technique’s feasibility [42]. The technique was successfully performed in 98.9% of gynecological patients by six different gynecologists in four centers. The median absolute amount of DNA was 2.23 μg. As a result, in 80% (24 of 30) of OC patients, specific mutations could be identified in the samples. The molecular analysis of uterine lavage holds great potential and significant promise for the earlier diagnosis of OC.

Deep sequencing was reported as the method of choice for detecting low-level signatures of tumor-derived mutations in liquid biopsies [63]. Duplex sequencing (DS) uses double-stranded molecular barcodes for error correction and decreases the error rate of sequencing from 10-3 to <10-7, so currently, it is the highest-accuracy sequencing NGS method. J. Salk in a 2018–2019 study demonstrated a high sensitivity (80%) of *TP53* mutation detection in OC patients’ uterine lavage using DS. However, low-frequency *TP53* mutations can be detected in healthy women without cancer. The *TP53* mutation rate progressively increased with age and shared the selection traits of clonal *TP53* mutations commonly found in human tumors. These results illustrate that in order to avoid false-positive results, the mutant allele frequency threshold should be used for careful differentiation of cancer-specific changes from age-associated mutations. The combined approach of uterine lavage and DS allows the collection of cancer cells very close to the anatomical site of the tumor’s origin, and an ultra-accurate DNA sequencing technology can detect exceptionally low-frequency mutations [41,64].

### 3.2. Circulating Tumor Cells

Circulating tumor cells (CTCs) are living tumor cells that are released into the bloodstream. They can be released either by primary tumor or metastases and very rarely can be found in healthy individuals. Various CTC isolation techniques were reported based either on physical (microfluidic platforms, density gradient centrifugation, and others) or biological characteristics (immunoaffinity based, immunomagnetic beads, and the functional cell adhesion molecule (CAM) uptake cell enrichment method). Immunohistochemistry (IHC), analysis by real-time PCR (RT-PCR), fluorescent in situ hybridization, and the detection of epithelial and mesenchymal markers are usually used for CTCs’ identification. Analysis of the CTC detection methods, the diagnostic and prognostic significance in OC by Du-Bois Asante et al. revealed that using only IHC for CTCs’ quantification had detection rates from 7.7 to 98%, while using RT-PCR had a 14–91% rate A combination of these two methods for the identification of CTCs had the detection rates ranging from 65 to 100% [65]. Pearl M. et al. demonstrated CTCs’ detection and isolation by using a CAM-based functional cell enrichment and identification platform and IHC from 129 OC patients before surgery. The detection rate was 88.6%, and the PPV (positive predictive value) for all stages was 97.3% with 83% sensitivity. The sensitivity for the detection of EOC stages I and II was 41.2%, the specificity 95.1% and the PPV 77.8% [55]). When qRT-PCR was added to the IHC for identification of CTCs in the next study, the detection rate increased up to 100% (*N* = 31/31) with the same specificity and sensitivity [54,55]. Zhang et al. performed CTC detection by immunomagnetic bead screening, targeting epithelial antigens on OC cells, in combination with a multiplex RT-PCR. In early-stage disease (IA-IB), the CTC detection rate was 93%, which compared favorably with the 64% of patients with elevated CA 125 levels [47]. While diagnostic techniques, such as the CAM uptake cell enrichment method for CTC isolation, RT-PCR, and qRT-PCR for CTC quantification have been a major addition to IHC and serum markers, molecular profiling has provided important information on genetic alterations linked to resistance to chemotherapy, which should in the future help clinical decision making.

### 3.3. Cell-Free DNA and Circulating Tumor DNA 

Cell-free DNA (cfDNA) comprises small DNA fragments that circulate freely in the bloodstream. In healthy individuals, cfDNA derives from apoptotic cells and can increase in the case of exercise, inflammation, or trauma. Recent studies confirmed that cancer patients have higher levels of cfDNA in the blood with an average of 180 ng/L (range from 0 to 1000 ng/mL), while healthy individuals or patients with benign ovarian pathologies have an average of 30 ng/mL (range from 0 to 100 ng/mL) [66,67,68]. Kamat et al. found elevated plasma cfDNA levels before surgery in OC patients compared to benign ovarian disease or healthy individuals, suggesting the use of cfDNA as a diagnostic and prognostic marker [69]. A meta-analysis of nine studies by Q. Zhou et al. evaluated the accuracy of cfDNA for the diagnosis of OC. The results showed unsatisfactory sensitivity at 70%, but acceptable specificity at 90% for the diagnosis of OC. Quantitative analysis of cfDNA can hardly be used as an independent diagnostic biomarker for OC detection, but the combination with OC-specific biomarkers detectable in cfDNA may be a promising tool [70].

Apart from evaluating quantitative changes, various investigators focused on qualitative changes, including somatic mutations, aberrant DNA methylation, and chromosomal abnormalities in circulating cell-free DNA. Plasma circulating tumor DNA (ctDNA) has the potential to serve as a minimally invasive diagnostic tool with the detection sensitivity ranging from 76 to 83% and a specificity of 55–95%. Some studies examined gene fusion and somatic copy number variations. Analysis of ctDNA by using NGS and digital polymerase chain reaction (dPCR) has the advantage of identifying alterations that are specific to the tumor. On the other hand, pre-identification of mutant gene targets is required. Most researchers performing ctDNA analysis in OC are currently focused on HGSOC patients, and targeting of mutant *TP53* has demonstrated high sensitivity (>75–100%) and specificity (>80%) [36,65,71,72]. According to our literature review, J. Phallen et al. achieved the highest—68%—detection rate of OC FIGO stages I–II with 100% specificity. Targeted error correction sequencing (TEC-Seq) and digital droplet PCR for ctDNA detection were used in this study. The analytical performance characteristics of TEC-Seq, as well as ultrasensitive direct evaluation of sequence changes in ctDNA using massively parallel sequencing suggested that it may be suitable for early-stage OC. A variety of experimental and bioinformatic aspects may contribute to the high specificity of the TEC-Seq method such as deep sequencing and others [36]. A limitation of this study was the small number of patients included. Cohen et al. used a commercial blood test called CancerSEEK to analyze circulating proteins and mutations in cell-free DNA. On the basis of this study, the ctDNA detection rate was 98% for OC patients, but the early-stage detection rate was only up to 38%. The sensitivity of this test for OC was 99% [35]. Investigators at Johns Hopkins Kimmel Cancer Center applied the PapSEEK test (assay for mutations in 18 genes and assay for aneuploidy) to fluids from Pap brush, Tao brush, and plasma ctDNA. There were 1002 healthy controls, 382 with endometrial cancer, and 245 OC patients analyzed. ctDNA was found in 43% of 83 OC patients with the available plasma sample. When both Pap brush and plasma samples were tested, the sensitivity for OC patients was at 63% for all stages and 54% for early stages with 100% specificity [34].

### 3.4. Circulating Small Noncoding RNAs

#### 3.4.1. sncRNAs Are a Large Group of RNA Molecules with Size below <200 nt That Have No Protein Coding Potency

Small noncoding RNAs (sncRNAs), such as microRNAs (miRNAs), transfer RNA-derived small RNAs (tsRNAs), and P-element induced wimpy testis (PIWI) interacting small RNAs (piRNAs), are a hot spot in the field of biomedical research due to their active involvement in the initiation and development of various malignancies. Relative stability, short length, association with the Argonaute (Ago) family of proteins, and in most cases, the downregulation or silencing of target gene expression are the main common features of all sncRNAs [73].

#### 3.4.2. PIWI-Interacting RNA

PIWI-interacting RNA (piRNAs) interact with PIWIs—germline-specific Ago family nuclear RNA-binding proteins—and form piRNA-induced silencing complexes (piRISCs). The latest data demonstrate the contribution of piRNAs and PIWI proteins to the main carcinogenesis events: cell proliferation, resisting cell death, genome instability, invasion, and metastasis. PIWIs are essential for germline tissues and gametogenesis. Due to their restricted expression in reproductive tissue and tumors, PIWIs are classified as cancer/testis antigens (CTA). They are considered as excellent objects for diagnostic/prognostic biomarkers and targeted therapies. piRNAs regulate mechanistic RNA-based inhibition of transposable elements in germlines. They can target nontransposable elements as well—such as protein-coding messenger RNAs (mRNAs) —and modulate their expression, not only in germlines, but also in somatic cells, by a mechanism similar to that of miRNAs. piRISCs contribute to cancer development and progression by promoting a stem-like state of cancer cells, or cancer stem cells. The expression of germline genes in cancer reflects the ectopic activation in somatic tissues of a naturally silenced developmental program managing the escape from cell death, immune circumvention, and invasiveness [73,74]. In gynecologic malignancies, the study of piRNA pathophysiological significance, expression levels, and diagnostic performance remains exploratory.

#### 3.4.3. Transfer RNA-Derived Small RNAs 

Transfer RNAs (tRNAS) are ncRNAs that deliver amino acids to ribosomes during protein biosynthesis. Transfer RNA-derived small RNAs (tsRNAs) are unique sequences generated in the nucleus and derived from tRNA precursors or mature molecules and can be divided into two main groups: (1) transfer RNA-derived RNA fragments (tRFs) and (2) tRNA halves (tiRNAs). The production of tiRNAs is induced by stress such as starvation, hypoxia, heat shock, viral infection, and others. tsRNAs’ functional mechanism remains largely unknown. They play important roles in carcinogenesis, and their stability and higher expression levels place them as ideal diagnostic and prognostic biomarkers and therapeutic targets as well. We found no data reporting the diagnostic performance in terms of specificity and sensitivity of tRFs and tiRNAs in gynecological malignancies [73].

#### 3.4.4. microRNA

The investigation of the microRNA (miRNA) class has received the most attention of all sncRNAs to date. At present, >2800 human mature miRNAs have been identified and are registered at miRbase Release 22 [75]. miRNAs are involved in post-transcriptional regulation of gene expression through their binding to a complementary target. Depending on the region they bind to, they can lead to suppression or degradation of the target. miRNAs, which are overexpressed and targeted to tumor-suppressive protein-coding transcripts, are classified as oncogenic or onco-miRs. Tumor-suppressive miRNAs are responsible for the downregulation of oncogenes and are usually lost in cancer. The expression rate of the same miRNA may be different depending on the biological substance being tested. After biogenesis, miRNAs are secreted from cells to a variety of body fluids, such as plasma/serum, urine and vaginal discharge, breast milk, and others. They are bound to specific proteins or high-density lipoproteins or packed in extracellular vesicles (EV), such as exosomes, to avoid RNase degradation, and as a result, they acquire high stability. Exosomes play an important role in the information exchange between cells, and cancer derived exosomes reflect the tumor-specific miRNA profile [73].

The regulatory roles of miRNAs have been demonstrated in tumorigenesis, cell differentiation, proliferation, and apoptosis. For example, exosomal miRNA-200a, miRNA-26a are reported to be involved in OC cell proliferation, while miR-21–3p, miR-125 b-5p, miR-181 d-5p, and miRNA-205 promote tumor invasion and metastasis. miRNA-125b was shown to inhibit angiogenesis, while miRNA-374a, miRNA-374, miRNA-622, and miRNA-223 were shown to participate in cisplatin resistance mechanisms. miRNAs have been reported to have faster biogenesis and activation rates and longer half-lives relative to mRNA and proteins, which make them suitable for OC diagnostics at early stages [76,77,78,79].

A number of studies dedicated to the diagnostic, prognostic, and therapeutic potential of circulating miRNAs in OC have been published during the last decade. The pioneering study by Taylor et al. in 2008 documented eight exosome miRNAs: miR-21, miR-141, miR-200a, miR-200b, miR-200c, miR-203, miR-205, and miR-214 were reported to be elevated in the serum of OC patients compared to normal controls. Quantitatively, the eight different investigated miRNAs were not significantly different in early and late OC stages [80]. Subsequently, several lines of evidence reported that serum miRNAs (miRNA-141, miRNA-200a, miRNA-200b, and miRNA-200c) were upregulated in OC patients compared to normal controls or borderline tumors [56,57]. Moreover, differences in miRNA-200c expression levels between OC stages might contribute to the cancer staging system, since more advanced tumors have lower miRNA-200c levels. On the contrary, the level of miRNA-141 displayed a trend towards increasing from FIGO stage I to stage IV [56]. Kim S. et al. analyzed seven serum exosomal miRNAs and reported that miRNA-141, 200a, and 200b were expressed at extremely low levels and qualified them as inappropriate serological biomarkers. Expression levels of miRNA-93, -145, and -200c were significantly more elevated in cancer tissues as compared to benign and borderline tumors. miRNA-145 was identified as the best-performing single marker with a sensitivity of 91.7% and accuracy of 86.8%. Even higher sensitivity (97.9%) was observed when miRNA-145 was combined with CA 125 assessment [60]. However, altered levels of miRNA-145, as well as some other widely investigated miRNAs (miRNA-21, miRNA-221, miRNA-155) were observed not only in OC, but in other malignancies as well [81]. Numerous studies reported the excellent behavior of the selected miRNAs or their combinations as biomarkers for OC, but did not investigate whether the profile of OC patients is distinct from those of other cancers, so it becomes clear that any single miRNA is unlikely to be a reliable biomarker. Yokoi et al. in 2017 performed miRNA sequencing to identify candidate miRNAs that could be useful in the early detection of OC and cancer subtype classification. They identified eight miRNAs, which were validated by qRT-PCR and statistical cross-validation with a large research cohort and were applied to determine the optimal combination of miRNAs. They succeeded in distinguishing early-stage OC from benign tumors with 86% sensitivity and 83% specificity and OC patients versus healthy controls with 92% and 91% respectively [58].

Later Yokoi et al. constructed three kinds of discrimination models: (1) OC vs. noncancer, (2) OC vs. other cancers  +  noncancer, and (3) OC vs. borderline/benign ovarian tumors  +  noncancer. A total of 4046 serum samples, including 333 ovarian cancers, 66 borderline ovarian tumors, 29 benign ovarian tumors, 2759 noncancer controls, and 859 other solid cancers, were analyzed by a miRNA microarray, yielding comprehensive miRNA expression profiles. This is the first large-scale comprehensive analysis of circulating miRNAs in OC, which identified promising miRNA combinations for early–stage detection of OC. Data revealed that selected combined miRNAs could be successfully used to discriminate OC from lung, gastric, breast, hepatic, colorectal, and pancreatic carcinoma, but not from sarcoma and esophageal squamous cell carcinoma. While using circulating miRNA profiles, a sensitivity of 99% and a specificity of 100% were observed discriminating between OC and noncancer patients, but discrimination was more difficult between OC and borderline or benign ovarian tumors [59].

### 3.5. Other Potential Biomarkers

#### 3.5.1. Long Noncoding RNAs 

Long noncoding RNAs (lncRNAs) are class of mRNA-like transcripts that are longer than 200 nucleotides. They lack a protein-coding ability and are involved in various biological roles. Similar to miRNAs, lncRNAs are frequently aberrantly expressed in different types of cancer. Few studies have examined lncRNAs or their combination with other circulating markers for colorectal, hepatocellular, and lung cancer detection and showed their diagnostic potential [82,83,84].

#### 3.5.2. Extracellular Vesicle-Associated Proteins

Extracellular vesicles (EVs) contain cell surface proteins, as well as miRNAs and other molecules. EV-associated proteins and lncRNAs were investigated as potential biomarkers and showed greater sensitivity comparing to conventional biomarkers, but there are no data about the value to OC patients [82,83,84,85].

#### 3.5.3. Tumor-Educated Platelets 

Tumor-educated platelets (TEPs) are known for their function as the main player in the systemic/local responses to tumor growth and their ability to change the RNA profile. Tumor-associated biomolecules are transferred to platelets, resulting in their “education”, and also, platelets are activated to induce specific splicing of premessenger RNAs (pre-mRNAs). TEPs may offer certain advantages, including their abundance and easy isolation, their high-quality RNAand their capacity to process RNA in response to external signals. In 2015, Best et al. first suggested the diagnostic potential of TEPs by mRNA sequencing. Their patient cohort included six tumor types: nonsmall-cell lung carcinoma, colorectal cancer, glioblastoma, pancreatic cancer, hepatobiliary cancer, and breast cancer. Their study revealed that TEPs can help to distinguish cancer patients from healthy individuals with a very high accuracy up to 96% and the primary tumor location with 71% accuracy [86]. Piek et al. in 2019 investigated TEPs as a diagnostic tool in differentiating OC FIGO stages I–II from benign ovarian tumors and reached 80% accuracy [87]. In the future, combinatorial analysis of TEPs with ctDNA/CTC can function as a potential blood-based source for cancer diagnostics. An ongoing clinical trial (NCT04022863), evaluating the accuracy of TEPs and ctDNA to determine the nature of an ovarian tumor, will bring more precise information on TEPs’ utility in OC detection [88].

## 4. Discussion

The early detection of OC, especially as early as FIGO stage II, appears critical to reduce mortality. The development of an efficient and cost-effective early OC screening test could be the path towards improved diagnostics for OC. This might lead to a substantial increase in patients who can undergo complete tumor resection and have less surgical complications due to smaller tumor burdens at the start. 

Liquid biopsy has an advantage over traditional biopsies in providing easy access and potentially additional biological information that might be useful for treatment decisions such as by the molecular analysis of a variety of material: CTC, ctDNA, and sncRNAs. 

A present limitation to assessing the value of liquid biopsies as a suitable diagnostic tool is the small number of patients enrolled in the studies, especially at early disease stages. Our review of the literature also showed significant variability in the terminology, in the timing and methodology of sampling, in the selection of gene panels assessed, in the methodology of isolation, and in the histological types of OC.

CTC: The impact of CTC isolation, detection, and molecular profiling and the cell capture technique selection for the purpose of early OC diagnosis is not clear. The detection rate of CTC in OC patients varies from 56–100% depending on the techniques [46,47,49,50,51,52,53,54,55]. Most studies evaluated CTC in advanced OC patients with only two studies reporting detection rates of 93% [47] and 41% [55] in early-stage disease. Presently, there is a high variability between platforms for the techniques of CTC isolation, and different IHC markers and/or gene panels are used for CTC content evaluation. Large blood samples are required. CTC surface markers suffer from a lack of tumor specificity. Only half of the studies reported on the diagnostic efficacy of the assay, and the sensitivity of the method was between 70 and 90% [46,50,51,54,55]. The feasibility of the technique in early-stage disease remains questionable.

ctDNA: Highly divergent detection rates of ctDNA in plasma samples have been reported, ranging from 38–100% and 35–68% for advanced and early-stage OC, respectively. The diagnostic performance of ctDNA can be evaluated via mutation and aberrant DNA methylation detection in selected genes (mainly *TP53*) or through the analysis of chromosomal abnormalities. The limitation of such a diagnostic approach could be met in occult OC patients with clinically and radiologically undetectable disease, a quite frequent situation in early HGSOC stages. Somatic mutations in selected genes, such as *TP53, KRAS, BRAF, PTEN,* or *PIK3CA,* are detectable in various human cancers and not specific for OC.

The application of NGS-based genetic or epigenetic panels offers a wide possibility to detect early OC-specific changes for cancer screening, but these NGS panels need to be developed and validated for clinical usage along with the standardization of sample collection and library preparation protocols. The development of protocols for tumor DNA collection from the uterine cavity and proximal tube lavage samples offers a new source of tumor-specific liquid biopsies collected from the sites very close to the primary tumor, confirming the likely origin as a gynecological cancer. Several ongoing clinical trials [83,84,86,87,89,90,91] evaluating the uterine lavage approach for OC detection are expected to clarify the most specific and sensitive biomarkers and molecular profiling methods.

miRNA: Despite the great clinical potential of miRNAs, it is also known that selected miRNAs are not specific for one tumor type. In most of the studies, combinations of miRNAs were analyzed with the aim to create specific discrimination models for disease detection and monitoring [57,58,59]. Before miRNAs may be considered as reliable biomarkers for clinical use, there are many issues to be solved, concerning the standardization of miRNA processing (from sample collection and storage to RNA isolation and data analyses) and large-scale validation. Most investigators used qRT-PCR for miRNA expression detection; however, this is a time-consuming and high-tech procedure, therefore not suitable for daily clinical testing. A less complicated, more rapid, more specific diagnostic test for OC is needed. To improve the biomarker sensitivity and specificity, further studies continue such as a national project in Japan, entitled the Development and Diagnostic Technology for Detection of miRNAs in Body fluids, which investigates serum miRNA profiles in 13 types of human cancers, including OC, which plans to include 10,000 patients. The aim of this study is to develop an algorithm allowing differentiating cancer from noncancer controls using expression levels of serum miRNA. Future research directions may also be highlighted. Despite the great clinical potential of miRNAs, the selection of OC-specific miRNAs remains a challenge due to the secretion of miRNAs from various cells, including blood cells.

## 5. Conclusions and Future Prospects

Innovative technologies based on very small samples are likely to drastically change medical practice in the near future. Presently available liquid biopsy assessments are not ready for use in clinical practice. Significant efforts remain to create reliable tests for early OC detection. Uterine lavage techniques are easy to apply and safe, and this approach appears very promising for implementation in daily clinical practice. miRNAs are promising biomarkers for cancer diagnosis and prognosis, and large-scale prospective clinical studies are ongoing. Research efforts directed toward single-cell analysis are likely to shed more light on diagnostic biomarkers and potential therapeutic targets in the future. 

## Figures and Tables

**Table 1:** Discriminatig features of major histotypes of epithelial ovarian cancer.

Histology	Cells of Origin	Precursors	More Frequent Somatic Mutations
Low-Grade Serous Carcinoma	Fallopian tube progenitor cell or secretory cell	Serous cystadenoma, adenofibroma, atypical proliferative serous tumor, noninvasive micropapillary serous borderline tumor	*KRAS* (30%), *BRAF* (30%), *NRAS, EIF1AX, USP9X, ERBB2, FRAR1, NF1, HRAS*
Mucinous Carcinoma	Unknown	Mucinous adenoma, mucinous borderline tumor	*CDKN2A (76%), KRAS and TP53 (both 64%), ERBB2 (26%), RNF43, BRAF, PIK3CA, ARID1A (8–12%)*
Endometrioid Carcinoma	Endometrial epithelial cells	Endometriosis and endometrial cell-like hyperplasia, endometrioid borderline tumor	*ARID1A* (30%), *PIK3CA* (30%), *TERT, CTNNB1*, *TP53*
Clear-Cell Carcinoma	Endometrial epithelial cells	Endometriosis, endometrioid borderline tumors	*PIK3CA* (50%), *ARID1A* (50%), *KRAS, MET, PTEN, CTNNB1, RPL22, TP53*
High-Grade Serous Carcinoma	Fallopian tube progenitor cell or secretory cell	SCOUT, *P53* signature, STIC	*TP53* (96–98%) *BRCA1/BRCA2* (10%, 25% somatic + germline); *CNAs* of *CCNE1* amplification, *PTEN* deletion, *RB1* and *NF1* loss
Carcinosarcomas	Unknown	Carcinomatous component	*TP53, CTNNB1*

STIC—serous tubal intraepithelial carcinomas, SCOUT—secretory cell OUT growth.

**Table 2:** Studies on ctDNA, DNA, CTC and microRNA in ovarian cancer.

Author (Year), References	Number of OC Patients	Specimen	Method	Genetic Marker/Antigen	Detection Rate (%)	Detection Rate (%) (I-II Stage)	Sensitivity (%)	Specificity (%)
K.K Lin et al. (2019) [33]	112 germline or somatic BRCA-mutant HGOC	Plasma (ctDNA)	Targeted-NGS	*BRCA1, BRCA2, TP53*	96 for *TP53*	NR	NR	NR
Y. Wang et al. (2018) [34]	83 OC	Plasma (ctDNA)	Pap SEEK-PCR-based error-reduction technology Safe-SeqS	18 genes + assay for aneuploidy	43	35	NR	100
Y. Wang et al. (2018) [34]	83 OC	Plasma (ctDNA) + Pap Brush samples	Pap SEEK-PCR-based error-reduction technology Safe-SeqS	18 genes + assay for aneuploidy	63	54	NR	100
P.A. Cohen et al. (2018) [35]	54 OC	Plasma (ctDNA) + proteins	CancerSEEK Targeted NGS	16 gene panel + 41 protein biomarkers	98	38	NR	>99 AUC = 0.91
J. Phallen et al. (2017) [36]	42 OC	Plasma (ctDNA)	Targeted NGS (TEC-Seq) and ddPCR	55 gene panel	71	68	NR	100
E. Pereira et al. (2015) [37]	22 HGSOC	Serum (ctDNA)	ddPCR, NGS, WES	*TP53, PTEN, PIK3CA, MET, KRAS, FBXW7, BRAF*	93.8	NR	81-91	60-99
A. Piskorz et al. (2016) [37]	18 OC	Plasma (ctDNA)	Targeted NGS	*TP53*	100	NR	NR	NR
R.C. Arend et al. (2018) [38]	14 OC	Plasma (cfDNA)	Targeted NGS	50 gene	100	NR	NR	NR
J.D. Cohen et al. (2016) [39]	32 HGSOC	Plasma cfDNA (instability)	WEG (WISECONDOR)	CNV	38	40.6	NR	93.8
A. Vanderst-ichele et al. [40]	57 OC and bordline tumors	Plasma cfDNA	WGS	CNV	67	NR	NR	99.6 AUC = 0.89
Y. Wang et al. (2018) [34]	245 OC	Cervix Pap brush samples (DNA)	Pap SEEK-PCR-based error-reduction technology Safe-SeqS,	18 genes + assay for aneuploidy	NR	33	34	99
		Tao Brush (DNA)	Pap SEEK-PCR-based error-reduction technology Safe-SeqS	18 genes + assay for aneuploidy	NR	45	47	100
Salk et al. (2019) [41]	10 OC	Uterine lavage (DNA)	Duplex Sequencing	*TP53*	80	NR	70	100
E.Maritschnegg (2018) [42]	33 OC	Uterine lavage (DNA)	Deep-sequencing	*AKT1*, *APC*, *BRAF*, *CDKN2A*, *CTNNB1*, *EGFR*, *FBXW7*, *FGFR2*, *KRAS*, *NRAS*, *PIK3CA*, *PIK3R1*, *POLE*, *PPP2R1A*, *PTEN*, *TP53*	80 for *TP53*	NR	NR	NR
E.Maritschnegg (2015) [43]	30 OC	Uterine lavage (DNA)	Massively parallel sequencing	*AKT1*, *APC*, *BRAF*, *CDKN2A*, *CTNNB1*, *EGFR*, *FBXW7*, *FGFR2*,	60 for *TP53*	100 for *TP53*	NR	NR
			With ddPCR and SafeSeqS	*KRAS*, *NRAS*, *PIK3CA*, *PIK3R1*, *POLE*, *PPP2R1A*, *PTEN*, *TP53*	80 for *TP53*			
B.K Erickson et al. (2014) [44]	5 OC	Vaginal tampon (DNA)	Massively parallel sequencing	NR	60	NR	60	NR
Kinde et al. (2013) [45]	22 OC	Liquid Pap smear tests (DNA)	Massively parallel sequencing	NR	41	NR	NR	NR
N. Li et al (2019) [46]	30 EOC	Plasma (CTC)	Magnetic nanospheres (MNs) + IHC	EpCAM, FRα	92	NR	75	90 AUC = 0.8
Zhang et al. (2018) [47]	109 EOC	Plasma (CTC)	Imunomagnetic beads (EpCAM, HER2 and MUC1) + multiplex RT-PCR	*EpCAM, HER2, MUC1, WT1, P16, PAX8*	90	93	NR	NR
Q Rao et al. (2017) [48]	23 EOC	Plasma (CTC)	Microfluidic system with immunomagnetic beads (EpCAM) + IHC	EpCAM, CK3-6H5, panCK	87	NR	NR	NR
M. Lee et al. (2017) [49]	54 EOC	Plasma (CTC)	Incorporating a nanoroughened microfluidic platform + IHC	EpCAM, TROP-2, EGFR, Vimentin, N-cadherin	98.1	NR	NR	NR
Dong Hoon Suh et al. (2017) [50]	87 EOC, bordline, benigh	Plasma (CTC)	Tapered-slit membrane filters + IHC	EpCAM, CK9	56.3	NR	77.4	55.8 AUC = 0.61–0.75
I. Chebouti et al. (2017) [51]	95 EOC	Plasma (CTC)	Adna Test Ovarian Cancer and EMT-1 Select/Detect + Multiplex RT-PCR	*EpCAM, ERCC1, MUC1, MUC16, PI3Ka, Akt-2, Twist*	82	NR	>90	>90
K. Kolostova et al. (2016) [52]	40 OC	Plasma (CTC)	MetaCell + IHC/qPCR	ICC: NucBlueTM, CelltrackerTM. *EpCAM, MUC1, MUC16, KRT18, KRT19, ERCC1, WT1*	58	NR	NR	NR
K. Kolostova et al (2015) [53]	118 OC	Plasma (CTC)	MetaCell + IHC/qPCR	ICC: NucBlueTM, CelltrackerTM. *EpCAM, MUC1, MUC16, KRT18, KRT19,*	65.2	NR	NR	NR
M. Pearl et al. (2015) [54]	31 EOC	Plasma (CTC)	CAM uptake-cell enrichment + IHC/RT-qPCR	EpCAM, Ca 125, CD44, seprase *EpCAM, CD44, MUC16, FAP*	100	NR	83	97
Pearl et al. (2014) [55]	129 EOC	Plasma (CTCs)	CAM uptake – cell enrichment + IHC	EpCAM, Ca 125, CD44, seprase	88. 6	41.2	83	95.1
Gao et al. (2015) [56]	143 all 74 EOC	Serum microRNA	qRT-PCR	miR-200c	NR	NR	72	70, AUC = 0.79
				miR-141			69	72, AUC = 0.75
Meng et al. (2016) [57]	163 EOC	Serum microRNA	TaqMan microRNA assays and ELISA	miR-200a	NR	NR	83	90, AUC = 0.91
				miR-200b			52	100, AUC = 0.81
				miR-200C			31	100, AUC = 0.65
				3miRNAs set			88	90, AUC = 0.92
Yokoi et al. in (2017) [58]	269 all 155EOC	Serum microRNA	qRT-PCR + statistical cross-validation methods	8 miRNA combination	NR	86	92	91, AUC = 0.96
Yokoi et al. in (2018) et al. [59]	EOC 333	Serum microRNA	Microarrays	10 miRNAs set miRNA-320a, -665, -1275, -3184-5p, -3185, -3195, -4459, 4640-5p, -6076, and -6717-5p. EOS vs. non cancer	NR	NR	99	100, AUC = 0.72–1.0
Kim S. (2019) [60]	68 all 39HGOC	Serum microRNA	qRT-PCR	miRNA-145	NR	NR	91.7	86.8, AUC = 86.8
				miRNA-200C			72.9	90.0, AUC = 77.9

NR: not reported; OC- ovarian cancer; EOC: epithelial ovarian cancer; ddPCR: Droplet digital PCR; RT-PCR: real time PCR technology; qRT-PCR: quantitative real time PCR; NGS: next generation sequencing; CAM: cell adhesion matrix; WES: whole exome sequencing; TGS: targeted gene sequences; HGSOC: high grade serous ovarian cancer; ddPCR: droplet digital PCR; AUC- areas under the ROC curves; IHC: immunocytochemistry staging; CNV: Copy number variation; WES: Whole exome sequencing; Safe-SeqS: Safe-sequencing system; WGS: Whole genome sequencing.
